# Rapid assessment of Hib disease burden in Vietnam

**DOI:** 10.1186/1471-2458-11-260

**Published:** 2011-04-25

**Authors:** Batmunkh Nyambat, Duc Anh Dang, Hien Anh Nguyen, Trinh Quynh Mai, Manju Rani, Mary PE Slack, Paul E Kilgore

**Affiliations:** 1 Division of Translational Research, International Vaccine Institute, Seoul, South Korea; 2 National Institute of Hygiene and Epidemiology, Hanoi, Vietnam; 3 Expanded Programme on Immunization (EPI), Western Pacific Regional Office, World Health Organization, Manila, Philippines; 4 Haemophilus Reference Unit, Respiratory and Systemic Infection Laboratory, Health Protection Agency Centre for Infections, Colindale, London, UK

**Keywords:** Hib, vaccine, RAT, disease burden

## Abstract

**Background:**

Several countries have applied the *Haemophilus influenzae *type b (Hib) rapid assessment tool (RAT) to estimate the burden of Hib disease where resources for hospital- or population-based surveillance are limited. In Vietnam, we used the Hib RAT to estimate the burden of Hib pneumonia and meningitis prior to Hib vaccine introduction.

**Methods:**

Laboratory, hospitalization and mortality data were collected for the period January 2004 through December 2005 from five representative hospitals. Based on the WHO Hib RAT protocol, standardized MS Excel spreadsheets were completed to generate meningitis and pneumonia case and death figures.

**Results:**

We found 35 to 77 Hib meningitis deaths and 441 to 957 Hib pneumonia deaths among children < 5 years of age annually in Vietnam. Overall, the incidence of Hib meningitis was estimated at 18/100,000 (95% confidence interval, CI, 15.1-21.6). The estimated Hib meningitis incidence in children < 5 years age was higher in Ho Chi Minh City (22.5/100,000 [95% CI, 18.4-27.5]) compared to Hanoi (9.8/100,000 [95% CI, 6.5-14.8]). The Hib RAT suggests that there are a total of 883 to 1,915 cases of Hib meningitis and 4,414 to 9,574 cases of Hib pneumonia per year in Vietnam.

**Conclusions:**

In Hanoi, the estimated incidence of Hib meningitis for children < 5 years of age was similar to that described in previous population-based studies of Hib meningitis conducted from 1999 through 2002. Results from the Hib RAT suggest that there is a substantial, yet unmeasured, disease burden associated with Hib pneumonia in Vietnamese children.

## Background

Prior to the introduction of universal childhood immunization with *Haemophilus influenzae *type b (Hib) vaccine, Hib was the most common cause of life-threatening bacterial infection (most often resulting in meningitis, bacterial pneumonia, and sepsis) in young children in industrialized countries [1]. *Haemophilus influenzae*, including Hib, is a well-known cause of childhood and adult disease with manifestations that include pneumonia, meningitis, epiglottitis, empyema, septicemia, septic arthritis, osteomyelitis, pericarditis, and cellulitis [2,3]. Globally, Hib is responsible for an estimated 3 million cases of serious disease and 386,000 childhood deaths every year [4]. Since the introduction of Hib conjugate vaccines in many countries, the decline in Hib disease burden has been documented in several countries where the Hib conjugate vaccine is in routine use [5-7]. To date, the Hib conjugate vaccine has been considered safe and highly effective [8].

Over the past decade, relatively few studies have documented the burden of Hib disease among Asian children [9,10]. This limited evidence has hindered the introduction of Hib vaccines in some countries. Thus, the assessment of Hib disease burden in children is an important pre-requisite for informing policymakers of the value of routine Hib immunization [11]. However, measuring Hib disease burden is not straightforward, particularly in countries with limited microbiological laboratory diagnostic capacity and antibiotic overuse. Although Hib meningitis exacts high costs from the health-care system and society due to its severity and associated long-term sequelae, it is believed that the majority of Hib disease in developing countries is manifested as pneumonia [12,13].

For many countries, local disease burden assessments may be obtained using Ministry of Health (MOH) surveillance data and a review of the local scientific literature. However, this strategy may not be possible where surveillance data is limited. In 2006, the Ministry of Health in Vietnam initiated consideration of Hib conjugate vaccine introduction into the national Expanded Programme on Immunization (EPI). To support decisions regarding the use of Hib vaccines in Vietnam, we conducted a rapid assessment of the Hib disease burden using a standardized rapid assessment tool (RAT) developed by WHO [14]. The objective of this study was to estimate the incidence of Hib meningitis and Hib pneumonia among children < 5 years of age in Vietnam. The results of this assessment will be compiled with data from other clinical, epidemiologic and laboratory studies of Hib disease to provide a more comprehensive picture of the Hib disease burden among children in Vietnam.

## Methods

Vietnam is located in Southeast Asia and has a total population estimated at 86 million persons [15]. Vietnam's public health sector is highly regarded globally, and it is renowned for an efficient public health system capable of delivering routine EPI vaccines at sustained high coverage rates. Hanoi and Ho Chi Minh City (Vietnam's major cities) have the largest urban populations in the country, and contain several major children's hospitals, which include Pediatric Hospital #1 and Pediatric Hospital #2 in Ho Chi Minh City, and National Pediatric Hospital, Bach Mai Hospital, and St. Paul Hospital in Hanoi (Table 1).

**Table 1:** Study hospital and population characteristics, Vietnam

Hospital name	Total Beds (Pediatric beds)	City	Population, # < 5 yrs^1^
Bach Mai	350 (30)	Hanoi	0-59 months: 217639

St. Paul	500 (138)		

National Pediatric	580		

Hospital #1	1000	HCMC	0-59 months: 406451

Hospital #2	1000		

^1^Population data from Vietnam national census office.

This study applied the Hib RAT, which was developed by the WHO, to estimate the Hib disease burden in Vietnam [16]. A retrospective analysis was conducted using existing data, specifically the numbers of meningitis and pneumonia cases and deaths in children < 5 years of age that may be attributable to Hib infection. The Hib RAT calculations utilize two methods for determining meningitis and pneumonia cases and deaths (the meningitis incidence rate method and the under-five mortality rate method). The meningitis incidence rate method uses local estimates of Hib meningitis incidence based on analysis of retrospective data from hospitals or from other local Hib studies in order to estimate the national burden of total Hib disease. The under-five mortality rate method uses the proportion of childhood deaths from acute lower respiratory infection (ALRI) attributable to Hib (13%) to the total number of under-five deaths due to ALRI, minus the neonatal deaths unlikely to be due to Hib infection [12]. Backward calculations are then done to estimate the total number of Hib pneumonia and meningitis cases, which are based on estimated case-fatality rates for Hib pneumonia and meningitis.

For data collection, the study team visited five major children's hospitals in Hanoi and Ho Chi Minh City. These hospitals were identified for the study by Vietnam's National Institute of Hygiene and Epidemiology (NIHE). These hospitals were selected because each had well-established clinical and microbiological facilities that were capable of assessing children with severe disease, including pneumonia, meningitis, and sepsis, as well as laboratory capability for the identification of *Haemophilus influenzae *[16]. In each of the hospitals, discharge records were inspected over a two-year period (January 2004 through December 2005) to identify any child less than five years of age who had a diagnosis of meningitis based on the International Classification of Diseases, 10^th ^Revision (*ICD*-10) diagnostic codes. After identification of children hospitalized with meningitis, a complete medical chart review was conducted to identify children with purulent meningitis characterized by abnormal cerebrospinal fluid (CSF). In addition to medical chart review, all microbiological records for the period from January 2004 through December 2005 were reviewed to identify invasive *Haemophilus influenzae *isolates from CSF or blood culture laboratory specimens. The study team also collected data on mortality rates among infants and children < 5 years of age from the Vietnam Ministry of Health in order to estimate the number of deaths associated with Hib [17].

Three Microsoft Excel worksheets from the standardized WHO Hib RAT protocol were used to enter information on the number of patients with purulent meningitis, number of patients with clinical meningitis who had a lumbar puncture performed, number of patients with Hib isolated in CSF, population of children < 5 years of age, previous estimates of Hib meningitis incidence, ratio of Hib pneumonia to Hib meningitis (5 to 1), and Hib meningitis and Hib pneumonia case fatality rates [14,16]. Using these inputs, the annual Hib meningitis incidence rate was calculated. Ninety-five percent confidence intervals (CIs) for incidence rates were calculated by the Wilson score method [18]. Two additional standardized worksheets were completed to estimate the total number of cases and deaths associated with both Hib meningitis and pneumonia. This study was approved by the International Vaccine Institute's Institutional Review Board (IRB) and the National Institute of Hygiene and Epidemiology's (NIHE) scientific and ethical review committee. Human experimentation guidelines of the authors' institution(s) were followed in the conduct of clinical research.

## Results

### Suspected, purulent, and confirmed bacterial and Hib meningitis

Based on available data from the three children's hospitals in Hanoi during the two-year study period from January 2004 through December 2005, we found 145 cases of suspected meningitis, 85 cases of purulent meningitis, including 17 cases with a bacterial pathogen identified, and 10 cases in which Hib was detected (Table 2). There were no in-hospital deaths due to Hib meningitis identified in the Hanoi hospitals. In Ho Chi Minh City, a total of 1,115 cases of suspected meningitis were found over the two-year study period. In addition, hospitalization and laboratory records showed that 404 children were hospitalized with purulent meningitis of which 34 children had a bacterial pathogen. Of these, Hib was identified from 15 (44.1%) patients. One death due to Hib meningitis was identified from the two children's hospitals in Ho Chi Minh City.

**Table 2:** Clinical & laboratory results from hospital record reviews in Hanoi and Ho Chi Minh City, Vietnam, January 2004 through December 2005

Case category	Hanoi	Ho Chi Minh City
Suspected meningitis	145	1115

Purulent CSF specimens, children < 5 years	85	404

Purulent meningitis cases in which a bacterial pathogen was identified	17	34

Purulent meningitis cases in which Hib was identified	10	15

Child deaths due to Hib meningitis	0	1

### Total estimated cases of Hib meningitis and Hib pneumonia

Using the standardized worksheet calculations for the "incidence rate" method, we derived a national total of 35 Hib meningitis deaths and 441 Hib pneumonia deaths. Using the alternative method (the "U5MR" method), a total of 77 Hib meningitis deaths and 957 Hib pneumonia deaths were estimated at the national level. In addition, these two methods estimated that there are a total of 883 to 1,915 cases of Hib meningitis and 4,414 to 9,574 cases of Hib pneumonia per year in Vietnam among children < 5 years of age. Using the standardized worksheet for the incidence estimation, the estimated Hib meningitis incidence among children < 5 years of age was 9.8/100,000 (95% CI, 6.5-14.8) in Hanoi and 22.5/100,000 (95% CI, 18.4-27.5) in Ho Chi Minh City. Overall, the incidence rate of Hib meningitis among children < 5 years of age in Vietnam was 18/100,000 (95% CI, 15.1-21.6).

## Discussion

This study demonstrates that Hib disease is an important disease among children < 5 years of age in Vietnam. Since 1998, six studies of Hib disease have been conducted at major hospitals in Vietnam [9,19-23]. In addition, national Vietnamese agencies have conducted prospective, population-based surveillance over a two-year period from 2000 to 2002 [9]. This study focused on acute Hib meningitis among children less than five years of age. The overall incidence rate of Hib meningitis was 12/100,000 children < 5 years of age. Given that this study only considered Hib meningitis, previous studies that determined the ratio of Hib pneumonia to Hib meningitis allowed us to estimate the total incidence of Hib disease (including Hib pneumonia would result in a total incidence rate of 60/100,000 in children < 5 years of age) [24,25].

This study has some limitations. In Vietnam, relatively few clinical hospital microbiology laboratories have the capacity for Hib culture and identification. This situation is slowly improving but Hib bacterial culture laboratory capacity is mostly restricted to large, national- or regional-level tertiary care hospitals located in major cities such as Hanoi and Ho Chi Minh City. As a result, data inputs for the Hib RAT were only obtained from large hospitals. More recently in Vietnam (June 2010), Hib vaccine was introduced as a component of a pentavalent vaccine into the routine immunization program. With this new vaccine introduction, efforts are underway to conduct routine hospital-based sentinel surveillance for Hib with improved laboratory diagnosis using CSF/blood culture, bacterial antigen detection, and nucleic acid testing (e.g., polymerase chain reaction). These surveillance efforts will be critical to understanding the future impact of Hib vaccines in Vietnam.

The diagnosis of Hib pneumonia is difficult even under the best of circumstances because a very high proportion of bacterial cultures are negative. For that reason, it is believed that the proportion of all-cause pneumonia attributable to Hib can be ascertained most accurately using the Hib vaccine as a probe [26]. In Hib vaccine probe studies, rates of pneumonia are compared between groups of children who receive or do not receive the Hib conjugate vaccine. The percentage reduction in Hib disease observed between these two groups provides indirect information on the proportion of pneumonia due to Hib. Knowledge of the burden of Hib pneumonia relative to Hib meningitis from previous Hib vaccine probe studies suggests that Hib pneumonia is five times more common compared to Hib meningitis [24,25]. Because these studies were limited to only two populations from the Gambia and Chile, it is arguable that this ratio may be different in other settings. Nevertheless, because there have been few Hib vaccine probe studies in which pneumonia was assessed in a standardized fashion, this ratio has been utilized in the Hib RAT to derive the total number of Hib disease cases and deaths. The range of Hib meningitis and pneumonia deaths found in Vietnam and other Hib RAT studies results from the application of two different estimation methods that utilize different assumptions, sources of data and formulae to derive the meningitis and pneumonia deaths figure [14,27].

Hib disease burden studies in developed countries are time-consuming and very expensive [28]. In less-developed countries, such studies pose unique challenges for existing technical capacities, logistics, and programmatic sustainability. The primary goal behind the Hib rapid disease burden assessment is to provide an easily attainable yet easy-to-implement estimate of the Hib burden of disease. The results of rapid disease burden assessment studies may suffer from perceptions of low credibility since the underlying assumptions of the rapid disease burden assessment tool are based on limited clinical, epidemiological or laboratory data. Nevertheless, rapid disease burden assessment tools have been used in a wide range of countries to enhance basic understanding of Hib disease.

In Vietnam, the Hib RAT data analysis was also used to derive the estimated incidence rates of Hib disease. Interestingly, the results of this analysis fall within the range of the 95% confidence intervals found in the prospective study of Hib and pneumococcal disease conducted in Khanh Hoa province in Vietnam from 2005-2006 with support from PneumoADIP [19]. In this study, the incidence of all Hib disease was found to be 22.9/100,000 (95% CI, 10.3-51.2) in children less than five years of age. Furthermore, these results also overlap with the incidence rate of Hib meningitis found in Hanoi, Vietnam [9]. The incidence rates for Hib disease in Vietnam are also consistent with those found in several European countries prior to conjugate Hib vaccine introduction into routine immunization programs [29]. Thus, conducting the Hib RAT assessment in Vietnam has allowed us to confirm findings from previous population-based incidence studies conducted in Vietnam. Furthermore, future use of the Hib RAT to periodically estimate the burden of Hib disease following Hib vaccine introduction may give national agencies an additional tool with which to assess the impact of Hib vaccination.

## Conclusion

Despite potential limitations of the Hib RAT in terms of data inputs from only a few laboratories, and making assumptions with respect to the ratio of Hib pneumonia to Hib meningitis, the Hib RAT appears to provide a valid approach to approximating the incidence rate and burden of Hib disease among children < 5 years of age. Previous to this study, the consistency of incidence rates derived from Hib RAT assessments and population-based studies conducted in the same country have never been shown. These encouraging results suggest that rapid disease burden assessment tools may be developed and validated for other diseases. Such rapid assessment tools may be particularly valuable in resource-limited settings where laboratory-based surveillance is challenging to sustain.

## Competing interests

The authors declare that they have no competing interests.

## Authors' contributions

BN and PEK conceived and designed the study, assisted with data collection, performed the data analyses and drafted the study manuscript. HAN and TGM implemented standardized methods for hospital data collection, verified data sources and accuracy and participated in writing of the study manuscript. DAD, MR and MPES provided input into data collection, reviewed outputs from data analysis and assisted in editing of the study manuscript. All authors read and approved the final manuscript.

## Pre-publication history

The pre-publication history for this paper can be accessed here:

http://www.biomedcentral.com/1471-2458/11/260/prepub
